# Purifying Selection, Density Blocking and Unnoticed Mitochondrial DNA Diversity in the Red Deer, *Cervus elaphus*

**DOI:** 10.1371/journal.pone.0163191

**Published:** 2016-09-20

**Authors:** Zbigniew Borowski, Magdalena Świsłocka, Maciej Matosiuk, Paweł Mirski, Kamil Krysiuk, Magdalena Czajkowska, Anetta Borkowska, Mirosław Ratkiewicz

**Affiliations:** 1 Department of Forest Ecology, Forest Research Institute, Raszyn, Poland; 2 Institute of Biology, University of Bialystok, Bialystok, Poland; Universita degli Studi di Roma La Sapienza, ITALY

## Abstract

The trajectories of postglacial range expansions, the occurrence of lineage patches and the formation and maintenance of secondary contact between lineages may mostly reflect neutral demographic processes, including density blocking, that may leave long-lasting genetic signatures. However, a few studies have recently shown that climate may also play a role. We used red deer, a large, mobile herbivore that is assumed to be sensitive to climate change, to test hypotheses of possible selection on the mitochondrial DNA cytochrome *b* gene (mtDNA cyt*b*) and competitive and/or density-blocking (using mtDNA control region). We searched for a possible link between the phylogeographic structure and abiotic climatic variables. Finally, we tested for isolation by distance and isolation by environment and assessed the impact of human-mediated translocations on the genetic structure of red deer. Our analysis of 30 red deer populations in Poland using the mtDNA control region (*N* = 357) and cytochrome *b* (*N* = 50) markers not only confirmed the presence of the Western and South-Eastern lineages of the species but also indicated the presence of a previously unnoticed, rare relic haplotype that grouped together *C*. *e*. *italicus* from Italy (the Mesola deer). No significant signs of positive selection were detected for the mtDNA cyt*b* gene in the studied red deer. However, a significant signal for purifying selection was found in our study that may explain the narrowness of the contact zone because gene flow between the Western and South-Eastern lineages should drive relatively strong mito-nuclear incompatibilities. MtDNA control region differentiation among red deer populations in Poland correlated with different abiotic climatic variables. Strikingly, the southernmost ice sheet limits during the Elsterian was the most important factor, and it explained the largest amount of variation. However, neither isolation by distance (IBD) nor isolation by environment (IBE) were recorded, and a very limited impact of human translocations was evident. The above-mentioned results suggest that in contemporary red deer populations in Poland, the phylogeographic pattern is well preserved, and long-term processes (density and/or competitive blocking) still play a major role.

## Introduction

Numerous population genetic studies have shown the presence of a clear phylogeographic structure within a variety of species in Europe [1–3]. This structure includes the occurrence of distinct mitochondrial DNA lineages of allopatric origins from separate glacial refugia and the formation of suture zones in which such lineages establish secondary contact [1, 4]. A few studies have recently shown that climate may play a role in shaping the phylogeographic structure [5–7] and the location/maintenance of suture zones [8]. A possible link between the phylogeographic structure and the average temperature in January was shown for the weasel *Mustela nivalis* [2] and the roe deer *Capreolus capreolus* [9]. The response of different species to past climatic changes is, however, not straightforward [4], and the abovementioned link observed for weasels was not found in the bank vole [10], although both species showed considerable similarities in their phylogeographic structures. Strikingly, the abovementioned studies did not analyse possible selection on coding protein sequences. Rare studies based on translated mitochondrial genes have shown that demographic history is a main source of the phylogeographic structure with several episodes of local adaptations to peculiar environments [11]. Recent mitogenomic phylogenetics of the bank vole revealed an excess of radical changes to the primary protein structure for geographically restricted clades from Italy and Norway [7] that may reflect a relatively stronger selective pressure at the latitudinal extremes of the species distribution.

Present-day phylogeographical patterns are often the result of more complex histories, as shown in very few ancient DNA studies [12]. For example, Meiri *et al*. [13] used red deer, a large herbivore, to test an expansion-contraction model. Using ancient mtDNA of the species, the authors [13] showed that at the end of the Last Glacial Maximum (LGM, at c. 16,000 cal. yr BP), the Western European lineage (termed “A” *sensu* Skog *et al*. [14]) expanded eastwards from the Iberian refugium to Central and Northern Europe. Concurrently, the South-Eastern lineage (“C”) experienced northwards colonization from the Balkans, South-Western Asia and possibly Italy. Meiri *et al*. [13] also confirmed that prior to the LGM, haplotypes belonging to the South-Eastern lineage reached as far as the UK and Spain. Based on these results, the authors [13] concluded that the red deer was sensitive to climate change. Thus, it is plausible to search for a link between the phylogeographic structure and the abiotic climatic factors and to test for possible selection acting on mtDNA in the contemporary red deer.

Possibly due to climate change, several red deer haplotypes belonging to the South-Eastern lineage went extinct, including those from Spain, Serbia and England [13]. Interestingly, the latter two haplotypes (from Serbia and England) grouped with *C*. *elaphus italicus* comprising a well-supported branch within the South-Eastern lineage [15]. This branch, termed the Mesola red deer, is native to the Italian peninsula and has recently been assigned to a subspecies *Cervus elaphus italicus* nova ssp. [16]. Its population is highly endangered and it is assumed to be the only remnant of this relic branch within the South-Eastern lineage. It cannot, however, be ruled out that a few rare haplotypes belonging to almost extinct branches are still present within the contemporary *C*. *elaphus* distribution.

The trajectories of postglacial range expansions, the occurrence of lineage patches and the formation and maintenance of secondary contact between lineages may mostly reflect neutral demographic processes, including density blocking that may leave long-lasting genetic signatures [17, 18]. If immigrants that arrived first to recently deglaciated areas experience demographic expansion, their high densities will lower the chances of secondary colonizers to incorporate into a gene pool of a recipient population. In effect, the patterns of genetic discontinuities would be visible across the range of the species, as well documented for European hedgehogs (e.g., [18]). It is not clear, however, if density blocking can operate in the case of large and thus mobile terrestrial mammals. The well-recognized mtDNA lineages of red deer and their origin and distribution in Europe [13, 14, 19], coupled with recent discoveries based on ancient DNA [13], makes the red deer, a large herbivore, an ideal system for testing hypotheses of density-blocking and possible selection on mtDNA. An area in Poland seems particularly interesting, where the orientation of the contact zone between the Western (A) and South-Eastern (C) lineages of this species is on the north-south axis [19]. This may again be associated with abiotic climatic factors, including glacial periods.

The population genetic structure of the red deer as an important game animal could have been blurred by numerous human-mediated introductions and translocations [20–22]. Thus, the biogeographic structure of this animal could have been affected, or alternatively, long-term processes (density blocking and/or selection) still play a major role. If the latter is the case, one should expect a clear phylogeographic pattern of the red deer throughout Poland, a narrow contact zone with little mtDNA introgression between the two lineages and a very limited impact of recent translocations. The sequence analysis of a large portion of the mtDNA control region in over 350 modern red deer specimens throughout Poland and a subset of samples for the complete cytochrome *b* gene allowed us to distinguish between long-term and contemporary processes.

In particular, we aimed to (1) assess a link between the phylogeographic structure of red deer and climate, (2) test for possible selection acting on the mitochondrial cytochrome *b* gene, (3) estimate the position and width of a contact zone between the lineages, (4) detect possible rare variants and (5) quantify the impact of contemporary processes, including isolation by distance, isolation by environment, and human interventions, on red deer genetic structure.

## Materials and Methods

### Samples and Laboratory Methods

**Fig 1:**
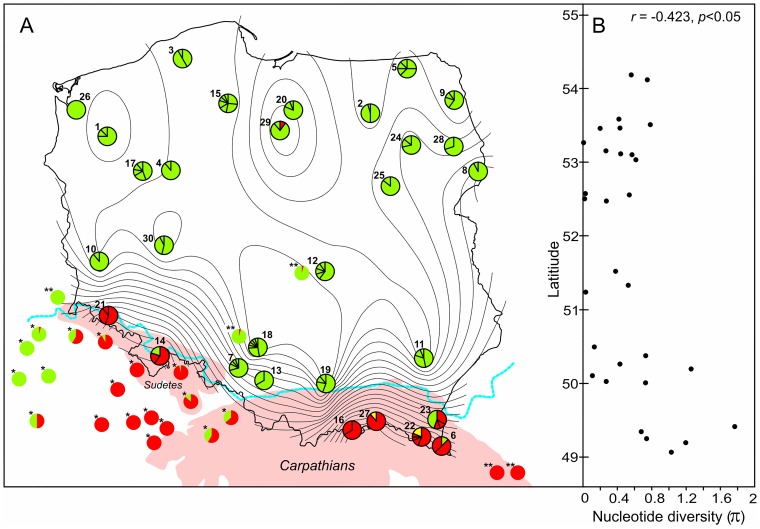
**(A) Pie diagrams indicate the frequency of cr mtDNA haplotypes belonging to the Western lineage (green), the Eastern lineage (red) and the Mesola lineage (yellow) of red deer.** PC1 values from principal component analysis (PCA) performed on a population pairwise matrix of *Φ*_ST_ values (black solid line) for cr mtDNA provided a synthetic genetic map that was superimposed onto a geographic map of Poland. The dotted blue line indicates the southernmost ice sheet limit (the Elsterian; Ehlers, *et al*. 2004) during the maximum extension in the Pleistocene. * Frequencies of cr mtDNA from Krojerová-Prokešová, *et al*. (2015). ** Occurrence of cr mtDNA control region haplotypes from Niedziałkowska *et al*. (2011). **(B)** Correlation latitude with nucleotide diversity (*π*) showing the width of the contact zone between two distinct cr mtDNA lineages (Western and South-Eastern) of red deer in Poland.

In the mitochondrial control region (cr mtDNA) study, we analysed 357 red deer samples from 30 populations in Poland (Fig 1A). The sample sizes from a given populations ranged from 7 to 25 individuals (Table 1). Muscle tissues were obtained from legally culled individuals during the hunting seasons in 2007 and 2008. Samples were stored at –20°C prior to DNA extraction with the DNeasy Blood and Tissue Kit (Qiagen, Hilden, Germany). PCR amplification of cr mtDNA was performed in a GeneAmp PCR System 9600 thermal cycler (Applied Biosystems) in a final volume of 5 μL containing ∼25 ng genomic DNA, 1.7 μL Qiagen multiplex PCR Master Mix (1x), 0.3 μL mix of the primers PRO and PHE [23] and 1 μL RNase-free water. The following PCR profile was used: an initial denaturation at 95°C for 15 min, followed by 35 cycles consisting of denaturation at 94°C for 30 s, annealing at 57°C for 90 s, extension at 72°C for 60 s, and final extension step at 60°C for 30 min. Amplified PCR products of cr mtDNA were purified with the CleanUp kit (A&A Biotechnology) and sequenced with the BigDye^™^ Terminator Cycle Sequencing Ready Reaction Kit v 3.1 (Applied Biosystems) in both the forward and reverse directions using the amplification primers. The cycling protocol for the sequencing reaction was as follows: 25 cycles consisting of 95°C for 20 s, 50°C for 15 s and 60°C for 1 min. Unincorporated dideoxynucleotides were eliminated from the sequencing reaction using the ExTerminator Kit (A&A Biotechnology). The sequencing products were run on an automated capillary sequencer ABI 3130 (Applied Biosystems). The resulting 750-bp-long sequences of the cr mtDNA were aligned in BioEdit v7.0.4 [24] and revised manually. In addition, the complete mtDNA cyt*b* gene (1,140 bp long) was amplified and sequenced in a chosen set of 50 red deer samples using primers ML103 and MH104 [25]. The selected set of 50 out of 357 red deer included individuals possessing all distinct cr mtDNA haplotypes identified in our study (S1 Table). The PCR and sequencing conditions for the mtDNA cyt*b* were identical to those used for the cr mtDNA. To ensure consistency of haplotypes, the amplification and sequencing of cr mtDNA and cyt*b* were repeated for 10% of the individuals as blank samples. The sequences of cr mtDNA and cyt*b* haplotypes were submitted to GenBank and assigned the following accession numbers: KX496901–KX496946 (S1 Table).

**Table 1:** Mitochondrial DNA control region diversity indices for 30 red deer populations studied in Poland.

No	Population	*N*	*Nh*	*h* (SE)	*π* (SE)	*S*	PD
1.	Bierzwnik	16	3	0.43 (0.14)	0.62 (±0.36)	12	4.63 (±2.40)
2.	Strzałowo	22	3	0.57 (0.05)	0.43 (±0.26)	7	3.23 (±1.73)
3.	Trzebielino	12	3	0.62 (0.09)	0.75 (±0.44)	11	2.62 (±2.90)
4.	Podanin	9	2	0.22 (0.17)	0.03 (±0.04)	1	0.22 (±0.29)
5.	Gołdap	8	5	0.86 (0.11)	0.56 (±0.35)	11	4.18 (±2.32)
6.	Lutowiska	8	3	0.68 (±0.12)	1.03 (±0.61)	25	7.71 (±4.02)
7.	Rudy	17	5	0.51 (±0.14)	0.11 (±0.09)	5	0.79 (±0.60)
8.	Białowieża	25	2	0.15 (±0.09)	0.02 (±0.03)	1	0.15 (±0.22)
9.	Głęboki Bród	10	4	0.64 (±0.15)	0.42 (±0.27)	7	3.16 (±1.78)
10.	Ruszów	9	2	0.22 (±0.17)	0.03 (±0.04)	1	0.22 (±0.29)
11.	Rudnik nad Sanem	15	4	0.69 (±0.08)	0.43 (±0.26)	8	3.18 (±1.74)
12.	Spała	10	5	0.67 (±0.16)	0.53 (±0.33)	11	3.93 (±2.15)
13.	Kobiór	9	2	0.50 (±0.13)	0.27 (±0.19)	4	2.00 (±1.24)
14.	Lądek Zdrój	10	3	0.62 (±0.14)	1.26 (±0.72)	25	9.42 (±4.73)
15.	Lubichowo	15	7	0.87 (±0.06)	0.78 (±0.44)	18	5.83 (±2.95)
16.	Piwniczna	9	2	0.50 (±0.13)	0.74 (±0.44)	11	5.50 (±2.92)
17.	Sarbia	9	5	0.81 (±0.12)	0.54 (±0.34)	8	4.06 (±2.23)
18.	Koszęcin	19	7	0.73 (±0.09)	0.73 (±0.41)	18	5.47 (±2.76)
19.	Niepołomice	9	4	0.69 (±0.15)	0.73 (±0.44)	15	5.44 (±2.89)
20.	Miłomłyn	16	3	0.34 (±0.14)	0.20 (±0.14)	7	1.52 (±0.96)
21.	Szklarska Poręba	9	3	0.64 (±0.13)	0.13 (±0.11)	3	1.00 (±0.74)
22.	Baligród	16	5	0.67 (±0.11)	1.19 (±0.65)	27	8.87 (±4.32)
23.	Bircza	9	4	0.75 (±0.11)	1.77 (±1.00)	25	13.22 (±6.58)
24.	Łomża	7	3	0.52 (±0.21)	0.57 (±0.37)	11	4.29 (±2.41)
25.	Ostrów Mazowiecka	7	2	0.29 (±0.20)	0.27 (±0.20)	7	2.00 (±1.28)
26.	Kiliniska	10	1	0.00 (±0.00)	0.00 (±0.00)	0	0.00 (±0.00)
27.	Łosie	9	2	0.22 (±0.17)	0.68 (±0.41)	23	5.11 (±2.73)
28.	Supraśl	10	2	0.47 (±0.13)	0.44 (±0.28)	7	3.27 (±1.83)
29.	Brodnica	10	3	0.38 (±0.18)	0.91 (±0.53)	30	6.78 (±3.49)
30.	Karczma Borowa	13	3	0.60 (±0.09)	0.38 (±0.24)	6	2.85 (±1.60)
	Total	357	35	0.90 (±0.01)	1.41 (±0.71)	55	10.57 (±4.83)

*N*–sample size; *Nh*–number of haplotypes; *h*–haplotype diversity; *π* –nucleotide diversity (%); *S*–number of segregating sites; PD–mean number of pairwise differences; SE–standard error.

### Molecular and Statistical Analyses

The levels of gene diversity of cr mtDNA were described within 30 red deer populations as the number (*Nh*) and frequency of haplotypes, haplotype diversity (*h*), nucleotide diversity (*π*, in %), the number of segregating sites (*S*) and the mean number of pairwise differences (PD). We examined directional patterns in cr mtDNA diversity throughout Poland as measured by the haplotype and nucleotide diversity in relation to latitude and longitude using STATISTICA6 (StatSoft, Tulsa, OK, USA). The diversity indices and pairwise *Φ*_ST_ and *F*_ST_ values for the 30 populations were calculated in ARLEQUIN v3.5.1.2 [26], and their significance was tested using 10,000 permutations that were corrected for multiple tests by Bonferroni correction. To assess whether patterns of genetic divergence were associated with geography, we applied the spatial AMOVA procedure using SAMOVA ver. 1.0 [27] and tested the significance of *Φ*- statistics using 10,000 permutations for *K* = 2 to *K* = 29 partitions of the sampling sites.

Principal component analysis (PCA) was performed based on a population pairwise matrix of *Φ*_ST_ and *F*_ST_ values for cr mtDNA data in GenAlEx v6.0 [28]. We then used PC1 scores with the geographic coordinates of 30 red deer populations for the spatial analysis of genetic differentiation, using the kriging algorithm in the Surfer 10 software (http://www.goldensoftware.com/demo-downloads). The contour lines of the first axis of the PCA were interpolated and superimposed onto a geographic map of Poland. By interpolating the PC1 scores of each population throughout the study area, we aimed to identify regions in which the genetic dissimilarity between populations was considerably higher than would be expected from the isolation-by-distance effect alone. Such geographic areas may represent contact zones between distinct cr mtDNA lineages (and could be revealed by *Φ*_ST_ values) or areas with strong genetic drift/founder effects (evaluated by *F*_ST_ values).

For cr mtDNA, we also performed an isolation by distance (IBD) test among all 30 populations studied using the software Isolation by Distance Web Service v3.23 ([29] http://ibdws.sdsu.edu/). We tested correlation coefficients between genetic distance [*F*_ST_/(1-*F*_ST_)] and the logarithm of geographical distance as recommended by Rousset [30]. We also determined whether isolation by distance was associated with landscape features by computing the correlation between the population-pairwise cr mtDNA genetic distance [*F*_ST_/(1-*F*_ST_)] values and GIS-based distance measures that vary as a result of costs arising from difficulties in moving through the landscape. As GIS-based distance measures, we used the length of the least-cost path corridors (LCP; ln-transformed), which were the most optimal dispersal routes for red deer and, second, the total cost of movement (ln-transformed) between each of the studied populations. We also used the least-cost path (LCP) algorithm to determine whether the studied populations were isolated by landscape features. Calculations of least-cost path lengths and cost-distances were computed using the Landscape Genetics toolbox [31] implemented in the ArcGIS 10.3 software. We used the Corine Land Cover 2012 raster dataset (European Environmental Agency, available at http://land.copernicus.eu/pan-european/corine-land-cover/clc-2012, accessed November 2015) to create a layer of friction in above-described software. Land cover types were reclassified to “costs” of movement by red deer according to Niedziałkowska *et al*. [21]. Deciduous and mixed forests were assumed to be the least costly to migrate over and were attributed cost– 1. Coniferous forests were attributed– 5, grasslands and arable lands– 30, wetlands– 50, scarce settlements– 70, dense settlements– 95 and water– 100 (see Niedziałkowska *et al*. [21] for details).

To identify correlations between cr mtDNA genetic distance values among red deer populations and abiotic climatic factors, we performed multivariate multiple regression analysis using DISTLM v5 [32] and DISTLM forward v1.3 [33]. The cr mtDNA population pairwise *Φ*_ST_ and *F*_ST_ values were used to construct the response matrices and tested against the following predictor matrices: latitude and longitude, average temperature in January, number of days with snow cover, average snow cover depth in January, number of frost days, annual rainfall and summer timing (in days). We also tested the impact of the Elsterian Glaciation and grouped our red deer populations within (1) or outside (0) of the southernmost ice sheet limit (the Elsterian; [34]) during the maximum extension in the Pleistocene (Fig 1A). Marginal tests of each predictor were performed, followed by conditional tests in which latitude and longitude were included as covariables of the predictor variables. Sequential tests were then performed using a forward selection procedure to produce a combined model of genetic differentiation in the red deer inhabiting Poland. The *p* values were obtained from 10,000 permutations. Abiotic climatic data were obtained from Lorenc [35].

To test the phylogenetic relationships among the concatenated mtDNA control region and cytochrome *b* haplotypes, we constructed phylogenetic trees using the Bayesian approach implemented in BEAST v1.7.2 [36] and a maximum-likelihood (ML) algorithm in Mega v5.05 [37] with 1,000 bootstrap replicates to assess the tree node support. We used additional mtDNA sequences of *C*. *elaphus* downloaded from GenBank (AB245427), *C*. *e*. *italicus* (KP859320 for cr mtDNA and KP859325 for mtDNA cyt*b*) and the sequence of the sika deer (*C*. *nippon*, AF016974) as outgroup. In the phylogenetic analyses, we used a nucleotide substitution model determined under the Akaike information criterion [38] implemented in jModelTest v0.1.1 [39]. The GTR+I+G model was selected as the best-fitting model for both cr and cyt*b* alignments independently, and it was used to construct Bayesian and ML trees from concatenated mtDNA sequences (1,890 bp). For our Bayesian analyses, we have previously used the Yule process tree [40]. The Bayesian analysis run consisted of an MCMC chain with 30,000,000 iterations sampled every 3,000th generation; the first 10% of the iterations were the burn-in. The sampled trees were summarized and annotated in TreeAnnotator v1.7.2 (BEAST software) and visualized in Figtree v1.3.1 (http://tree.bio.ed.ac.uk/software/figtree). The relationships among all detected concatenated control regions and cytochrome *b* of Polish red deer mtDNA haplotypes were also calculated and visualized by constructing a haplotype network using the median-joining method available in Network version 4.6.1.3 (http://www.fluxus-engineering.com). To construct a median-joining network, apart from the haplotypes found in our study, we used the *C*. *e*. *italicus* haplotypes previously published by Zachos *et al*. [16] under the following GenBank accession numbers: KP859320 (cr mtDNA) and KP859325 (mtDNA cyt*b*).

We used the branch-site model A [41] of codon evolution that is available in the PAML package [42] to assess sites under positive selection in the mtDNA cyt*b* gene of the most abundant *C*. *elaphus* lineages in Poland (Western and South-Eastern lineage as foreground branches) based on the unrooted phylogeny (S1 Fig). The null hypothesis assumes that all branches that evolve under negative selection or neutrality are in opposition to the alternative hypothesis, in which the foreground branch is evolving under positive selection. Likelihood ratio tests (LRTs) were subsequently conducted to test the significance of the detection of sites under positive selection. We also employed the codon-based Z-test of selection implemented in MEGA v5.05 to infer purifying selection effects on the cyt*b* gene of red deer lineages in Poland.

## Results

Our alignment of the cr mtDNA (750 bp) of the 357 red deer from 30 populations in Poland yielded 55 polymorphic sites with a total of 46 transitions, 4 transversions and 5 indels, defining 35 cr mtDNA haplotypes. Twenty-three cr mtDNA haplotypes were previously unreported (for detailed information see S1 Table). Alignment of a whole cyt*b* gene (1,140 bp) in a selected group of 50 red deer provided 11 haplotypes. Three out of eleven cyt*b* red deer haplotypes were detected for the first time (for detailed information see S1 Table). Eleven cyt*b* gene haplotypes had 33 synonymous mutations, while 5 (at positions: 115, 352, 883, 898 and 1066) resulted in amino acid substitutions in the cyt*b* protein sequence (codons: 39, 118, 295, 300 and 356). Branch-site tests of positive selection performed in CODEML indicated that three sites occurred in the cyt*b* gene of the *C*. *elaphus* Western lineage (codons 39, 300 and 356), which may have been under positive selection. However, the subsequent likelihood ratio test did not confirm the significance of this finding. Moreover, the genetic diversity of the cyt*b* gene in red deer populations was affected by purifying selection, as indicated by the codon-based Z-test of selection (*Z* = 3.84, *p* < 0.001).

**Fig 2:**
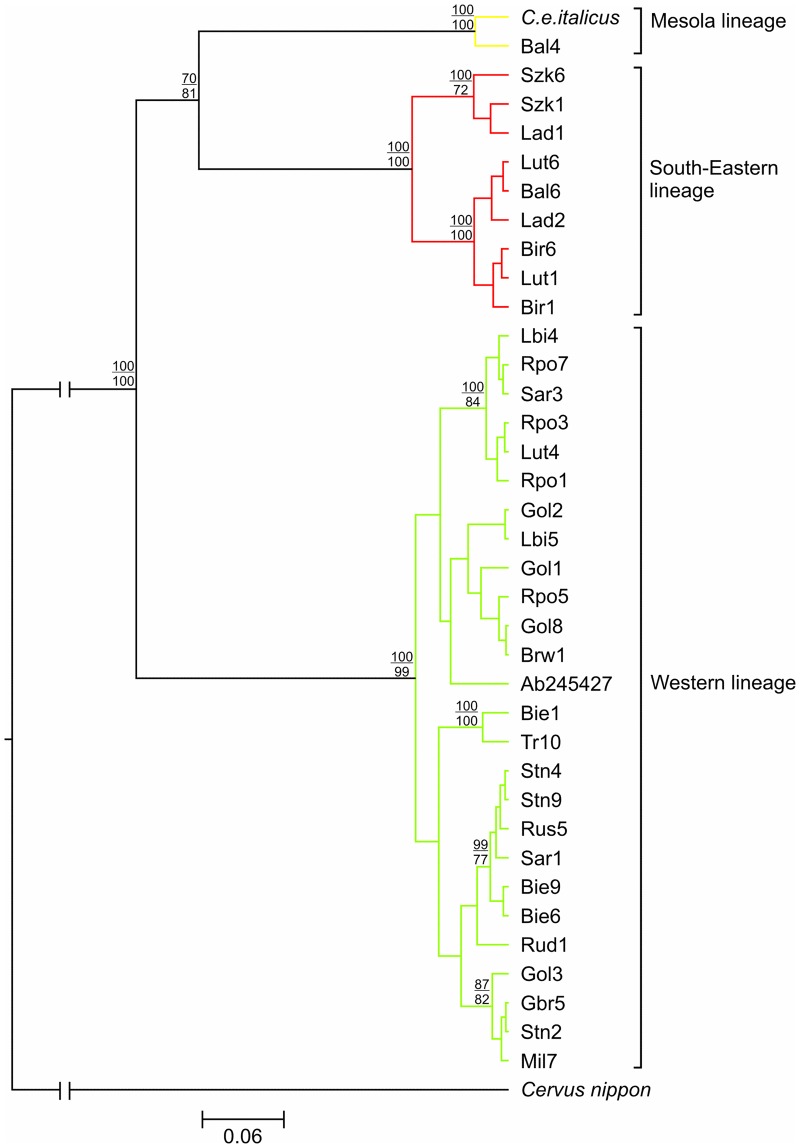
Bayesian tree computed with the GTR+I+G model of sequence evolution, representing the phylogenetic relationships among the concatenated control region and cytochrome *b* mtDNA sequences found in red deer samples from Poland. Maximum-likelihood topology computed with the GTR+I+G model of substitution, which was identical to the Bayesian tree.

Bayesian and maximum-likelihood phylogenetic reconstructions based on concatenated mtDNA control region and cytochrome *b* sequences (1,890 bp in total) produced strongly concordant topologies (Fig 2). The mtDNA trees revealed that all but one haplotype belonged to the following two main, highly supported lineages: the Western (A cf. Skog *et al*. [14]) and the South-Eastern (C). One of the haplotypes detected in Poland (Bal4) grouped together with *C*. *e*. *italicus* (the Mesola red deer described by Zachos *et al*. [16]). These two haplotypes formed a third lineage (termed by our group as “Mesola lineage”) that clustered together with the South-Eastern red deer lineage on the phylogenetic tree with high bootstrap support (Fig 2). Twenty-five haplotypes (found in 71.4% of individuals) detected in our study belonged to the Western lineage of the red deer, the other nine haplotypes (25.7% of individuals) to the South-Eastern lineage, while only one haplotype (present in 2.9% of individuals) belonged to a relic Mesola lineage. Thus, all haplotypes detected in Poland belonged to the Western clade of red deer and corresponded to *Cervus elaphus sensu stricto* [15].

**Fig 3:**
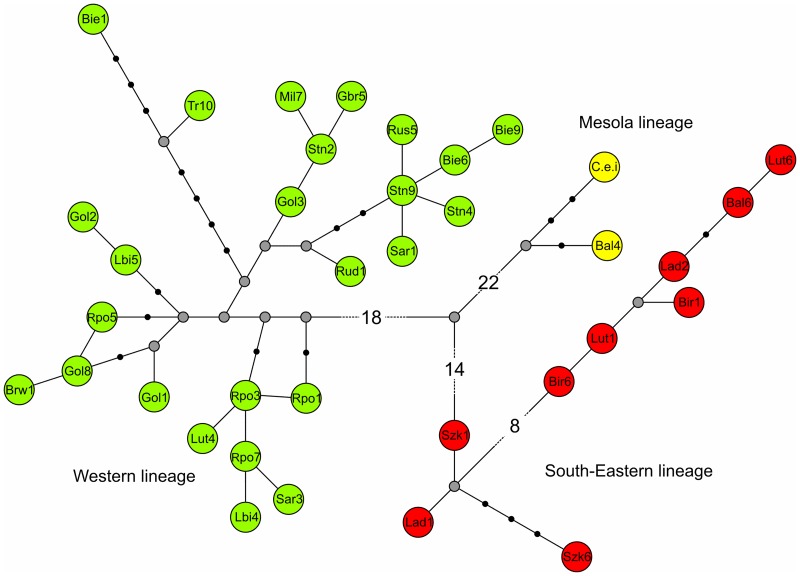
Median-joining network of mtDNA haplotypes from Poland belonging to the Western lineage, the South-Eastern lineage and the Mesola lineage. The network is based on an analysis of the concatenated control region and the cytochrome *b* sequences. Thirty-five haplotypes found in this study have a three letter code with a number, while the haplotype of *C*. *e*. *italicus* downloaded from GenBank is indicated by “C.e.i”. Missing haplotypes are indicated by a grey dot.

A median-joining network, based on concatenated cr mtDNA and cyt*b* sequences from this study and *C*. *e*. *italicus* [16], not only suggested the presence of three phylogenetic lineages of red deer in Poland, i.e., the Western, South-Eastern and Mesola lineages, but also their trichotomy (Fig 3). Haplotypes from the Western lineage differed by at least 34 substitutions from haplotypes belonging to the South-Eastern lineage. At least 38 and 44 mutations distinguished haplotype Bal4 (the Mesola lineage) from haplotypes belonging to the South-Eastern and Western lineages, respectively.

The analysis of cr mtDNA haplotypes identified in Poland showed that the Western lineage (A) was widely distributed throughout most of the country, apart from the southern regions, where haplotypes of the South-Eastern lineage predominated. The only exception was one individual from the South-Eastern lineage found in northern Poland (in population no 29), which was most probably due to translocation events. Translocations within the Western lineage that were also visible as the Gol8 haplotype present in population nos. 13 and 14 (southern Poland) were also found in the north-eastern part of the country (in population nos. 5, 8 & 28; S2 Table). The rare Bal4 haplotype representing the third relic lineage was found in south-eastern Poland in two populations only (Fig 1A & S2 Table). Twenty-two populations possessed haplotypes exclusively from the Western lineage, while as few as two populations were characterized by the presence of red deer belonging to the South-Eastern lineage only. Three populations (10%) exhibited the presence of individuals from both the Western and South-Eastern lineages (Fig 1A).

Among the 357 studied red deer individuals, the haplotype (*h*) and nucleotide diversity (*π*), as well as the pairwise difference (PD) values for cr mtDNA were relatively high (0.90, 1.40% and 10.51, respectively; Table 1). The measures of cr mtDNA diversity (*h* and *π*) did not show a significant relationship with the longitudinal position of the populations (haplotype diversity: *r* = 0.190, *ns*; nucleotide diversity: *r* = 0.286, *ns*). The correlation with latitude was not significant for haplotype diversity (*r* = -0.159, *ns*), but it was negative and significant for nucleotide diversity (*r* = -0.423, *p* < 0.05).

Pairwise genetic differentiation values for cr mtDNA among all studied red deer populations ranged from 0 to 0.98 (*Φ*_ST_) and from 0 to 0.89 (*F*_ST_), and in most cases (88.3% for *Φ*_ST_ and 91.1% for *F*_ST_ values, respectively) were significantly different from zero (S3 Table). The average *Φ*_ST_ and *F*_ST_ values for all studied populations were 0.54 and 0.40, respectively, indicating very high and significant (*p* < 0.001) overall structuring of red deer populations in Poland. SAMOVA analysis for cr mtDNA showed that the data were best explained assuming two or four groups of red deer populations (for *K* = 2: *Φ*_CT_ = 0.667, *p* < 0.001; *Φ*_SC_ = 0.437, *p* < 0.001 and *Φ*_ST_ = 0.813, *p* < 0.001, while for *K* = 4: *Φ*_CT_ = 0.680, *p* < 0.001; *Φ*_SC_ = 0.384, *p* < 0.001 and *Φ*_ST_ = 0.803, *p* < 0.001). The grouping with *K* = 2 perfectly reflected the division into A and C lineages, while when *K* = 4 was assumed, some additional structuring was found within southern red deer populations. Group 1 consisted of all red deer that belonged to the Western lineage, group two included populations no 6, 16, 22 and 27 situated in the south-eastern Poland, group three consisted of an admixed population no 23 from the same area while the group four formed two south-western Polish populations (no 14 and 21, see Fig 1). This configuration maximized the among-group variation (67.96%) and minimized the variation among populations within groups (12.29%) and within populations (19.74%). Synthetic genetic maps for the first axis (PC1) of the PCA (obtained from both *Φ*_ST_ and *F*_ST_ cr mtDNA values) superimposed onto a geographic map of Poland were in general similar and showed an acute gradient in the whole southern part of the study area (Fig 1A). These clines corresponded to the location of the contact zone between the Western and South-Eastern mtDNA lineages of red deer in Poland. Analysis of the nucleotide diversity values that were highest between latitudes 49° and 50° suggested the presence of an approximately 110-km-wide contact zone between the Western and South-Eastern lineages of red deer (Fig 1B).

Analysis of the isolation by distance pattern showed that genetic differentiation (measured using the linearized cr mtDNA *F*_ST_) did not increase significantly with geographical distance (*r* = 0.159; *ns*). No significant correlations were obtained when the linear *F*_ST_ values were correlated with the length of the least-cost path corridors (LCP; *r* = 0.1193; *ns*) or the total cost of movement (*r* = 0.110; *ns*) between the studied red deer populations.

The multivariate regression analysis performed in DISTLM showed that the chosen set of abiotic variables together explained a significant proportion of the multivariate variation in the red deer cr mtDNA *Φ*_ST_ data (62.0%, *p* < 0.001), but not for *F*_ST_ (39.9%, *ns*). Tests of the influence of geographical location on genetic differentiation among the red deer populations showed that pairwise cr mtDNA *Φ*_ST_ values correlated with latitude (20.5% of the explained variation, *p* < 0.001), but not with longitude (Table 2). In addition, the southernmost ice sheet limits during the Elsterian, number of days with snow cover, snow cover depth, number of frost days, average temperature in January, annual rainfall and summer timing as revealed by marginal tests, had significant effects on mtDNA differentiation among red deer populations in Poland (Table 2). When geographical coordinates were incorporated as covariables into the multiple regression analysis (conditional tests), all of the abovementioned abiotic factors again correlated with the pairwise *Φ*_ST_ values. In conditional tests, the cr mtDNA *F*_ST_ parameter correlated with the number of frost days, summer timing and ice sheet limits during the Elsterian between the red deer sampling locations (S4 Table). Sequential tests showed that the southernmost ice sheet limits during the Elsterian and longitude were the only significant factors. It is noteworthy that the former factor was the most important because it explained the largest amount of variation and was significant in marginal, conditional and sequential tests (Table 2 & S4 Table).

**Table 2:** Effects of abiotic climatic factors on genetic differentiation (as measured by *Φ*_ST_ values) among 30 red deer populations in Poland based on cr mtDNA.

Factor	Marginal tests		Conditional tests		Sequential tests	
	% var	*P*	% var	*P*	% var	*P*
Latitude	20.5	<0.001	-	-	2.5	ns
Longitude	7.1	ns	-	-	6.6	<0.05
Snow cover depth	19.6	<0.001	13.9	<0.001	1.5	ns
Days with snow cover	20.3	<0.001	15.1	<0.001	3.6	ns
Frost days	13.7	<0.01	16.8	<0.001	3.4	ns
Temperature in January	12.9	<0.01	11.1	<0.001	nd	-
Annual rainfall	29.3	<0.001	12.5	<0.001	1.7	ns
Summer timing	36.8	<0.001	21.1	<0.001	1.5	ns
The Elsterian	41.5	<0.001	24.3	<0.001	41.5	<0.001

% var–percentage of genetic variation explained by the particular variable; P–probability values; ns–non significant; nd–the test not done because one of the variables (temperature in January) did not increase the regression SS in the conditional test; the Elsterian means the southernmost ice sheet limit during the maximum extension in the Pleistocene.

## Discussion

### Phylogenetic Analyses and Detection of a Relic Lineage

The phylogenetic analysis based on the concatenated control region and cytochrome *b* mtDNA sequences was confirmed to be a powerful tool for disentangling intraspecific lineages, the contact zones among them and genetic structuring within a variety of mammalian taxa in Europe [1–3], as shown herein for the Western red deer, *C*. *elaphus*. Using contemporary samples, we documented the presence of the following two main mtDNA lineages in the studied species in Poland: Western and South-Eastern (A and C, respectively cf. Skog *et al*. [14]). No haplotypes belonging to the Sardinian/African lineage (B) were found within our study area, which is consistent with previous phylogeographical studies [14]. In addition, in two populations from south-eastern Poland, we demonstrated the presence of a distinct haplotype that grouped together with *C*. *e*. *italicus* from Italy (the Mesola deer [16]). In their phylogenetic study, Lorenzini & Garofalo [15] provided support for the subspecific status of the Mesola deer and postulated that it may represent a refugial lineage of eastern origin. Indeed, the haplotype network based on pairwise differences between the mtDNA haplotypes of red deer in our study clearly suggests a Western/South-Eastern/Mesola trichotomy (Fig 3). This trichotomy was also observed in the mitogenome analysis of these lineages (Matosiuk *et al*., unpublished). The abovementioned result strengthens the hypothesis regarding the unique characteristics and subspecific status of the Mesola deer [16] that is native to the Italian Peninsula. The inclusion of this haplotype into the ancient mtDNA analysis [13] revealed, however, that this haplotype belongs to a highly supported branch within the South-Eastern lineage, including ancient samples from Serbia and England. Our finding of a rare, relic haplotype (Bal4) in south-eastern Poland may also suggest that within the present-day red deer range, there could be more unnoticed mtDNA diversity than previously assumed. The occurrence of three lineages and the high nucleotide diversity in south-eastern Poland show that the Carpathians are an important area of intraspecific biodiversity for red deer, as also shown for the bank vole [10], roe deer [9] and several other species, although their role as a refugium for the species under study [43] was previously discounted [13, 19].

### Phylogeography, Evidence for Purifying Selection, Impact of Abiotic Factors and the Role of Density-Dependent Processes

Our analyses of cr mtDNA in the red deer showed that the majority of Poland is occupied by the Western lineage, while the South-Eastern one and the relic of the Mesola lineage represented by the Bal4 haplotype are restricted to the mountainous areas in southern Poland (Fig 1A). This observation agrees well with the concept of postglacial expansion of the Western lineage (A) from the Iberian refugium and subsequent colonization of Western and Central Europe at c. 16,000 cal. yr BP, as inferred from the radiocarbon dates [13] and genetic studies of contemporary samples [14, 19]. Concurrently, the South-Eastern lineage (C) expanded its range northwards from the Balkans [13] and crossed the Carpathians [14]. The eastward expansion from Iberia and northward expansion from the Balkans resulted in the formation of the contact zone between the Western and South-Eastern lineages of the red deer in southern Poland. Given that the species has a large dispersal potential [44], this zone is relatively narrow (approximately 110 km; Fig 1A and 1B), which could be explained by several factors that are not mutually exclusive. First, in southern Poland, a significant and abrupt decrease in the forest cover occurred in areas north of the mountainous areas (S2 Fig). While high forest cover has increased gene flow among red deer populations [21], low forest cover and the low mean temperature in January were the most important factors limiting red deer numbers in Poland [45]. Thus, low forest cover might be a significant barrier to the dispersal of red deer, albeit no isolation by environment in the contemporary red deer found in our study contradicted this assumption.

Natural selection could be another factor responsible for the observed phylogeographic pattern. The results of branch-site tests did not indicate significant signs of positive selection. However, a significant signal of purifying selection found in our study may explain the narrowness of the contact zone and suggests that possible gene flow between the Western and South-Eastern lineages should drive relatively strong mito-nuclear incompatibilities affecting individual fitness. Very low genetic exchange between red deer belonging to the Western and South-Eastern lineages was also evident. As few as 10% of the populations admixed, no mtDNA introgression tails were detected, and there was a scarcity of successful translocations between different mtDNA lineages, strengthening the hypothesis of purifying selection and consequent mito-nuclear incompatibilities. In addition, it is very likely that the European red deer, as a large herbivore, is susceptible to abiotic climatic factors [13, 45]. Our multiple regression analyses performed in DISTLM and DISTLM*forward* strongly support this hypothesis and corroborate the idea of “*competitive exclusion of secondary dispersers*” [17, 18]. Such exclusion during rapid postglacial demographic expansion might reflect fitness differences between lineages [17].

Of note, Waters *et al*. [18] suggested that founders can exclude latecomers by high density blocking rather than by selective advantage. Thus, density blocking may also explain the contemporary phylogeographic picture of red deer in Central Eastern Europe, with the Western lineage predominating in deglaciated areas of western, central and northern Europe [14]. In addition, we found the haplotype (Lut6) of the South-Eastern lineage in a single red deer within the area occupied by the Western lineage in one out of 30 populations, far from its natural distribution in the Carpathians Mountains (populations nos. 6, 16, 22, 23 & 27; S2 Table). A similar situation has also been observed in the Spała population ([19]; see also Fig 1A). The scarcity of such findings and narrowness of the hybrid zone does indicate the failure of dispersers (immigrant females in the case of mtDNA) to contribute genetically to recipient populations and may indicate a *density blocking* process despite the recorded high rate of gene flow observed among red deer populations in the seminatural landscape of Central Europe (e.g., north-eastern Poland; [21]).

In addition, Waters [17] noticed that “*competitive interactions between (…) lineages are likely to play a key role maintaining biogeographic disjunctions associated with historic barriers*, *even in the face of high dispersal potential*”. Indeed, multivariate regression analyses performed in DISTLM*forward* showed that a contemporary boundary between the Western and South-Eastern lineages of red deer corresponded well to the southernmost limits of the Elsterian Glaciation in Poland that reached the Sudetes and the Carpathians. This factor explained most of the observed variation and remained significant in all tests. Contemporary restriction of the South-Eastern lineage to the mountainous areas in Poland (and southern parts of Europe) can thus be explained in the following way. Prior to the LGM, the South-Eastern lineage was much more widespread and was present as far west as Spain, Belgium and England [13]. Subsequently, both lineages survived the last glacial period in separate refugia. After the LGM, the Western lineage rapidly expanded eastwards into deglaciated areas, while the northwards expansion of the Eastern lineage was slowed to a certain extent by the Carpathians and the Sudetes, and the Alps imposed a barrier for spatial expansion [14]. In effect, high densities of red deer from the Western lineage in the lowlands of Poland effectively blocked secondary dispersers from the South-Eastern lineage. Noticeably, the northward expansion of the latter lineage was successful in areas of low density or the absence of the Western lineage (Lithuania, Belarus and Western Russia; [19]). Thus, the phylogeographic structure of red deer and the maintenance of biogeographic disjunction in Central Europe is well explained by postglacial expansion and density-dependent processes.

### Effects of Translocations versus Phylogeographical and Population Genetic Structures

The red deer is an important game animal that experienced extensive introductions and translocations ([21, 22], see also numerous citations within these papers) that could have blurred their genetic structure. However, the translocations did not strongly affect the phylogeographic pattern of red deer in Central Eastern Europe ([21, 46], this study). This is clearly apparent in the almost complete absence of red deer possessing haplotypes of the South-Eastern lineage in Polish lowlands (see Fig 1A). It may indicate that translocations between red deer from different lineages were relatively rare and/or ultimately fruitless due to density and/or competitive blocking [17]. The translocations were, however, more apparent on local scales [14]. We found that the haplotype Gol8 (which belongs to the Western lineage) was very common in population no 8 (Białowieża, NE Poland), and it also occurred in two populations from south-western Poland (no 13 & 14; Fig 1A). This result provides evidence for eastward translocations and shows the origin (from southern Poland) of the reintroduced red deer in Białowieża (and in NE Poland in general) that took place in the late 19th and at the beginning of the 20th century ([21] and numerous citations therein). However, the postulated impact of translocations that potentially occurred in southern Poland on the genetic structure of the population of red deer in the northern Czech Republic [46] does not seem to be the case. Rather, natural immigration of the Western lineage through the Moravian Gate across the Polish-Czech border occurred (Fig 1A). The translocations and reintroductions in Poland probably resulted in the absence of a correlation between the haplotype diversity and the longitude and latitude. The same phenomenon was observed for nucleotide diversity and longitude. The only significant and negative correlation was found between nucleotide diversity and latitude, likely resulting from the presence of three lineages and contact zone between the Western and South-Eastern lineages in southern Poland rather than the latitudinal cline. Despite great genetic differentiation, as measured by the *Φ*_ST_ and *F*_ST_ values for cr mtDNA, neither isolation by distance (IBD) nor isolation by environment (IBE) were recorded. The least cost path (LCP) and the total cost of movement analyses did not reveal any link with genetic distances for contemporary red deer populations, even though these relationships were clearly visible in NE Poland using microsatellite data [21]. Thus, our results, including the SAMOVA analysis, clearly suggested that the phylogeographic pattern of red deer in Poland is well preserved in contemporary populations. It reflects postglacial expansion of two lineages from separate refugia and density and/or competitive blocking [17] between lineages. To our knowledge, it is the first study that documents density blocking and purifying selection between intraspecific lineages for a large, mobile terrestrial herbivore.

## Supporting Information

S1 Fig Neighbour-joining tree of the cytochrome *b* gene haplotypes of the chosen *Cervidae* and two Ruminants (*B*. *taurus* and *A*. *americana*) used in tests of selection on the cytochrome *b* gene. Numbers listed at nodes represent percent support for that node from 1,000 bootstrap replicates.
(TIF)

S2 Fig Least-cost paths (white lines) between the studied red deer populations. Migration costs are represented by friction values derived from land cover data.
(TIF)

S1 Table Mitochondrial DNA control region (cr mtDNA) and the respective cytochrome *b* (cyt*b*) haplotypes found in the red deer populations in Poland, their frequencies in the whole sample and the GenBank accession numbers for these haplotypes. The GenBank accession numbers for red deer obtained in this study are indicated in bold.
(DOCX)

S2 Table Frequency of 35 cr mtDNA haplotypes found in the red deer populations studied in Poland. (DOCX)

S3 Table Genetic differentiation of cr mtDNA between red deer population pairs in Poland, as measured by *Φ*_ST_ (below the diagonal) and *F*_ST_ (above the diagonal). Significant values are indicated in bold.
(DOCX)

S4 Table Effects of abiotic climatic factors on genetic differentiation (as measured by *F*_ST_ values) among 30 red deer populations in Poland based on cr mtDNA. (DOCX)
