# Supplementary figures and images for: Purifying Selection, Density Blocking and Unnoticed Mitochondrial DNA Diversity in the Red Deer, Cervus elaphus
Source: PLoS One. 2016 Sep 20;11(9):e0163191. doi: 10.1371/journal.pone.0163191 (PMC5029925; doi:10.1371/journal.pone.0163191)

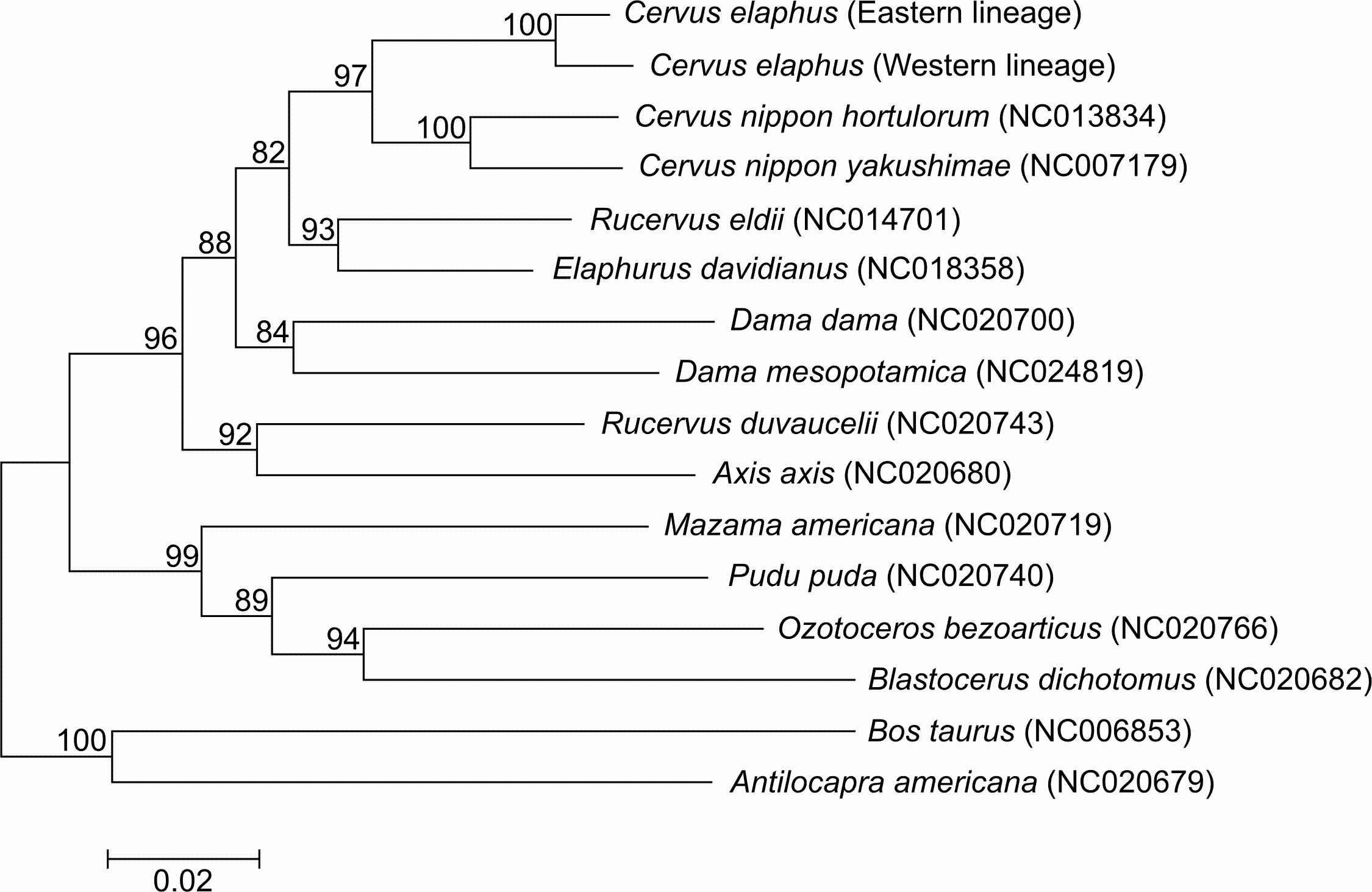

Supplement: S1 Fig — Numbers listed at nodes represent percent support for that node from 1,000 bootstrap replicates. (TIF) [file pone.0163191.s001.tif]

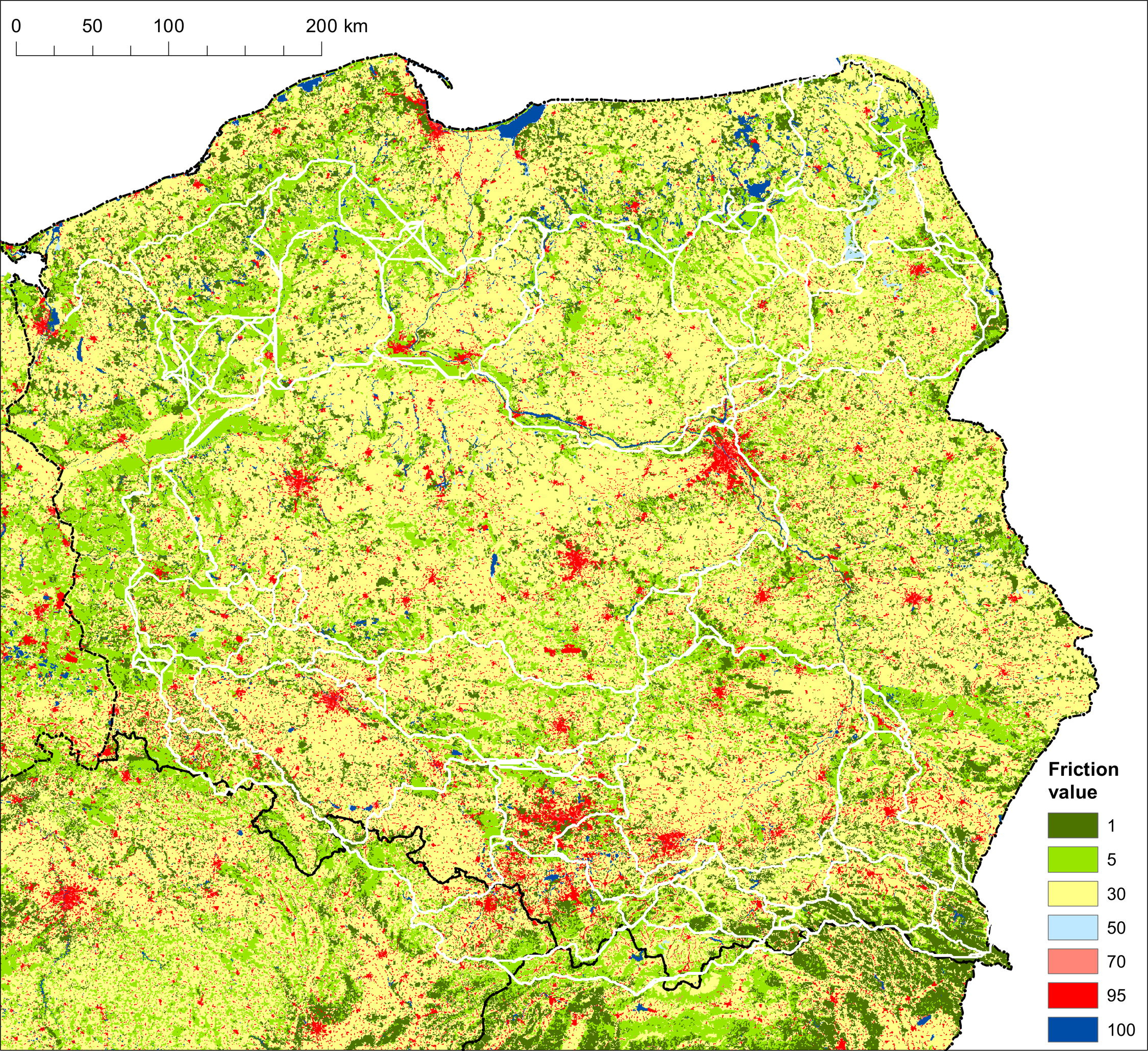

Supplement: S2 Fig — Migration costs are represented by friction values derived from land cover data. (TIF) [file pone.0163191.s002.tif]
