# Supplementary material for: Purifying Selection, Density Blocking and Unnoticed Mitochondrial DNA Diversity in the Red Deer, Cervus elaphus
Source: PLoS One. 2016 Sep 20;11(9):e0163191. doi: 10.1371/journal.pone.0163191 (PMC5029925; doi:10.1371/journal.pone.0163191)
Supplement: S1 Table — The GenBank accession numbers for red deer obtained in this study are indicated in bold. (DOCX) [file pone.0163191.s003.docx]

**S1 Table. Mitochondrial DNA control region (cr mtDNA) and the respective cytochrome *b* (cyt*b*) haplotypes found in the red deer populations in Poland, their frequencies in the whole sample and the GenBank accession numbers for these haplotypes.**

| No | cr mtDNA haplotype | GenBank number for cr mtDNA haplotype | cr mtDNA haplotype frequency (%) | cyt *b* mtDNA haplotype | GenBank number for cyt *b* mtDNA haplotype |
| --- | --- | --- | --- | --- | --- |
| 1. | Bal4 | **KX496935** | 1.1 | cyt*b-*11 | **KX496946** |
| 2. | Bal6 | **KX496932** KM410133* | 0.6 | cyt*b-*8 | **KX496943** AF489279‡ |
| 3. | Bie1 | **KX496901** | 11.8 | cyt*b-*1 | **KX496936** |
| 4. | Bie6 | **KX496902** | 1.1 | cyt*b-*2 | **KX496937** AB021099** |
| 5. | Bie9 | **KX496903** | 1.1 | cyt*b-*2 | **KX496937** AB021099** |
| 6. | Bir1 | **KX496933** EU436776 | 0.3 | cyt*b-*8 | **KX496943** AF489279‡ |
| 7. | Bir6 | **KX496934** | 0.3 | cyt*b-*8 | **KX496943** AF489279‡ |
| 8. | Brw1 | **KX496919** | 0.6 | cyt*b-*4 | **KX496939** KC562167 |
| 9. | Gbr5 | **KX496920** | 0.3 | cyt*b-*2 | **KX496937** AB021099** |
| 10. | Gol1 | **KX496907** KM410119* | 1.4 | cyt*b-*3 | **KX496938** KM410139* |
| 11. | Gol2 | **KX496908** | 0.3 | cyt*b-*6 | **KX496941** AY044858‡ |
| 12. | Gol3 | **KX496909** | 0.6 | cyt*b-*2 | **KX496937** AB021099** |
| 13. | Gol8 | **KX496910** KM410120* KM410130* | 10.9 | cyt*b-*4 | **KX496939** KC562167 |
| 14. | Lad1 | **KX496928** EU436793  EU436819  KM410110* KP859321† | 3.6 | cyt*b-*10 | **KX496945** KC181347 |
| 15. | Lad2 | **KX496929** EU436789 EU436795 JF893541* KM410107* KM410109* KM410115* | 0.6 | cyt*b-*8 | **KX496943** AF489279‡ |
| 16. | Lbi4 | **KX496923** | 2.0 | cyt*b-*5 | **KX496940** KM410143* |
| 17. | Lbi5 | **KX496924** KM410099* | 0.8 | cyt*b-*6 | **KX496941** AY044858‡ |
| 18. | Lut1 | **KX496926** EU436765 EU436766 EU436784 EU436801 | 1.4 | cyt*b-*8 | **KX496943** AF489279‡ |
| 19. | Lut4 | **KX496925** | 0.8 | cyt*b-*5 | **KX496940** KM410143* |
| 20. | Lut6 | **KX496927** KM410104* | 8.4 | cyt*b-*8 | **KX496943** AF489279‡ |
| 21. | Mil7 | **KX496918** | 0.3 | cyt*b-*2 | **KX496937** AB021099** |
| 22. | Rpo1 | **KX496911** | 0.6 | cyt*b-*5 | **KX496940** KM410143* |
| 23. | Rpo3 | **KX496912** KM410105* KM410112* | 10.6 | cyt*b-*5 | **KX496940** KM410143* |
| 24. | Rpo5 | **KX496913** | 0.3 | cyt*b-*4 | **KX496939** KC562167 |
| 25. | Rpo7 | **KX496914** | 2.7 | cyt*b-*5 | **KX496940** KM410143* |
| 26. | Rud1 | **KX496922** | 2.7 | cyt*b-*2 | **KX496937** AB021099** |
| 27. | Rus5 | **KX496921** | 0.3 | cyt*b-*2 | **KX496937** AB021099** |
| 28. | Sar1 | **KX496916** | 0.6 | cyt*b-*2 | **KX496937** AB021099** |
| 29. | Sar3 | **KX496917** | 0.3 | cyt*b-*5 | **KX496940** KM410143* |
| 30. | Stn2 | **KX496904** | 7.8 | cyt*b-*2 | **KX496937** AB021099** |
| 31. | Stn4 | **KX496915** | 1.1 | cyt*b-*2 | **KX496937** AB021099** |
| 32. | Stn9 | **KX496905** KM410131* | 22.7 | cyt*b-*2 | **KX496937** AB021099** |
| 33. | Szk1 | **KX496930** | 1.4 | cyt*b-*7 | **KX496942** AY070225‡ |
| 34. | Szk6 | **KX496931** KM410106* | 0.3 | cyt*b-*9 | **KX496944** |
| 35. | Tr10 | **KX496906** | 0.3 | cyt*b-*2 | **KX496937** AB021099** |

The GenBank accession numbers for red deer obtained in this study are indicated in bold.

* Krojerová-Prokešová J, Barančeková M, Koubek P. Admixture of Eastern and Western European red deer lineages as a result of postglacial recolonization of the Czech Republic (Central Europe). J Hered. 2015; esv018.

** Kuwayama R, Ozawa T. Phylogenetic relationships among European red deer, wapiti, and sika deer inferred from mitochondrial DNA sequences. Mol Phylogenet Evol. 2000; 15: 115–123.

† Lorenzini R, Garofalo L. Insights into the evolutionary history of *Cervus* (Cervidae, tribe Cervini) based on Bayesian analysis of mitochondrial marker sequences, with first indications for a new species. J Zool Syst Evol Res. 2015; 53: 340–349.

‡ Ludt CJ, Schroeder W, Rottmann O, Kuehn R. Mitochondrial DNA phylogeography of red deer (*Cervus elaphus*). Mol Phylogenet Evol. 2004; 31: 1064–1083.
