# Supplementary material for: Purifying Selection, Density Blocking and Unnoticed Mitochondrial DNA Diversity in the Red Deer, Cervus elaphus
Source: PLoS One. 2016 Sep 20;11(9):e0163191. doi: 10.1371/journal.pone.0163191 (PMC5029925; doi:10.1371/journal.pone.0163191)
Supplement: S2 Table — (DOCX) [file pone.0163191.s004.docx]

**S2 Table. Frequency of 35 cr mtDNA haplotypes found in the red deer populations studied in Poland.**

| **Population Haplotype** | **1** | **2** | **3** | **4** | **5** | **6** | **7** | **8** | **9** | **10** | **11** | **12** | **13** | **14** | **15** | **16** | **17** | **18** | **19** | **20** | **21** | **22** | **23** | **24** | **25** | **26** | **27** | **28** | **29** | **30** |
| --- | --- | --- | --- | --- | --- | --- | --- | --- | --- | --- | --- | --- | --- | --- | --- | --- | --- | --- | --- | --- | --- | --- | --- | --- | --- | --- | --- | --- | --- | --- |
| **Bal4** | **0** | **0** | **0** | **0** | **0** | **0** | **0** | **0** | **0** | **0** | **0** | **0** | **0** | **0** | **0** | **0** | **0** | **0** | **0** | **0** | **0** | **18.8** | **0** | **0** | **0** | **0** | **11.1** | **0** | **0** | **0** |
| **Bal6** | **0** | **0** | **0** | **0** | **0** | **0** | **0** | **0** | **0** | **0** | **0** | **0** | **0** | **0** | **0** | **0** | **0** | **0** | **0** | **0** | **0** | **12.5** | **0** | **0** | **0** | **0** | **0** | **0** | **0** | **0** |
| **Bie1** | **75.0** | **0** | **41.7** | **0** | **0** | **0** | **0** | **0** | **0** | **0** | **0** | **0** | **0** | **0** | **26.7** | **0** | **0** | **26.2** | **22.2** | **0** | **0** | **0** | **0** | **71.4** | **14.3** | **0** | **0** | **0** | **80.0** | **0** |
| **Bie6** | **12.5** | **0** | **0** | **0** | **0** | **0** | **0** | **0** | **0** | **0** | **0** | **0** | **0** | **0** | **6.7** | **0** | **0** | **5.3** | **0** | **0** | **0** | **0** | **0** | **0** | **0** | **0** | **0** | **0** | **0** | **0** |
| **Bie9** | **12.5** | **0** | **0** | **0** | **0** | **0** | **0** | **0** | **0** | **0** | **0** | **0** | **0** | **0** | **0** | **0** | **0** | **5.3** | **11.1** | **0** | **0** | **0** | **0** | **0** | **0** | **0** | **0** | **0** | **0** | **0** |
| **Bir1** | **0** | **0** | **0** | **0** | **0** | **0** | **0** | **0** | **0** | **0** | **0** | **0** | **0** | **0** | **0** | **0** | **0** | **0** | **0** | **0** | **0** | **0** | **11.1** | **0** | **0** | **0** | **0** | **0** | **0** | **0** |
| **Bir6** | **0** | **0** | **0** | **0** | **0** | **0** | **0** | **0** | **0** | **0** | **0** | **0** | **0** | **0** | **0** | **0** | **0** | **0** | **0** | **0** | **0** | **0** | **11.1** | **0** | **0** | **0** | **0** | **0** | **0** | **0** |
| **Brw1** | **0** | **0** | **0** | **0** | **0** | **0** | **0** | **8.0** | **0** | **0** | **0** | **0** | **0** | **0** | **0** | **0** | **0** | **0** | **0** | **0** | **0** | **0** | **0** | **0** | **0** | **0** | **0** | **0** | **0** | **0** |
| **Gbr5** | **0** | **0** | **0** | **0** | **0** | **0** | **0** | **0** | **10.0** | **0** | **0** | **0** | **0** | **0** | **0** | **0** | **0** | **0** | **0** | **0** | **0** | **0** | **0** | **0** | **0** | **0** | **0** | **0** | **0** | **0** |
| **Gol1** | **0** | **0** | **0** | **0** | **37.5** | **0** | **0** | **0** | **0** | **0** | **0** | **0** | **0** | **0** | **13.3** | **0** | **0** | **0** | **0** | **0** | **0** | **0** | **0** | **0** | **0** | **0** | **0** | **0** | **0** | **0** |
| **Gol2** | **0** | **0** | **0** | **0** | **12.5** | **0** | **0** | **0** | **0** | **0** | **0** | **0** | **0** | **0** | **0** | **0** | **0** | **0** | **0** | **0** | **0** | **0** | **0** | **0** | **0** | **0** | **0** | **0** | **0** | **0** |
| **Gol3** | **0** | **0** | **0** | **0** | **12.5** | **0** | **0** | **0** | **0** | **0** | **0** | **0** | **0** | **0** | **0** | **0** | **0** | **0** | **0** | **0** | **0** | **0** | **0** | **14.3** | **0** | **0** | **0** | **0** | **0** | **0** |
| **Gol8** | **0** | **0** | **0** | **0** | **12.5** | **0** | **0** | **92.0** | **0** | **0** | **0** | **0** | **66.7** | **20.0** | **0** | **0** | **0** | **0** | **0** | **0** | **0** | **0** | **0** | **0** | **0** | **0** | **0** | **70.0** | **0** | **0** |
| **Lad1** | **0** | **0** | **0** | **0** | **0** | **0** | **0** | **0** | **0** | **0** | **0** | **0** | **0** | **60.0** | **0** | **33.3** | **0** | **0** | **0** | **0** | **33.3** | **6.3** | **0** | **0** | **0** | **0** | **0** | **0** | **0** | **0** |
| **Lad2** | **0** | **0** | **0** | **0** | **0** | **0** | **0** | **0** | **0** | **0** | **0** | **0** | **0** | **20.0** | **0** | **0** | **0** | **0** | **0** | **0** | **0** | **0** | **0** | **0** | **0** | **0** | **0** | **0** | **0** | **0** |
| **Lbi4** | **0** | **0** | **0** | **0** | **0** | **0** | **0** | **0** | **0** | **0** | **0** | **0** | **0** | **0** | **6.7** | **0** | **44.4** | **0** | **11.1** | **0** | **0** | **0** | **0** | **0** | **0** | **0** | **0** | **0** | **10.0** | **0** |
| **Lbi5** | **0** | **0** | **0** | **0** | **0** | **0** | **0** | **0** | **0** | **0** | **0** | **0** | **0** | **0** | **13.2** | **0** | **0** | **5.3** | **0** | **0** | **0** | **0** | **0** | **0** | **0** | **0** | **0** | **0** | **0** | **0** |
| **Lut1** | **0** | **0** | **0** | **0** | **0** | **50.0** | **0** | **0** | **0** | **0** | **0** | **0** | **0** | **0** | **0** | **0** | **0** | **0** | **0** | **0** | **0** | **6.3** | **0** | **0** | **0** | **0** | **0** | **0** | **0** | **0** |
| **Lut4** | **0** | **0** | **0** | **0** | **0** | **12.5** | **5.9** | **0** | **0** | **0** | **6.7** | **0** | **0** | **0** | **0** | **0** | **0** | **0** | **0** | **0** | **0** | **0** | **0** | **0** | **0** | **0** | **0** | **0** | **0** | **0** |
| **Lut6** | **0** | **0** | **0** | **0** | **0** | **37.5** | **0** | **0** | **0** | **0** | **0** | **0** | **0** | **0** | **0** | **66.7** | **0** | **0** | **0** | **0** | **0** | **56.1** | **33.3** | **0** | **0** | **0** | **88.9** | **0** | **10.0** | **0** |
| **Mil7** | **0** | **0** | **0** | **0** | **0** | **0** | **0** | **0** | **0** | **0** | **0** | **0** | **0** | **0** | **0** | **0** | **0** | **0** | **0** | **6.3** | **0** | **0** | **0** | **0** | **0** | **0** | **0** | **0** | **0** | **0** |
| **Rpo1** | **0** | **0** | **0** | **0** | **0** | **0** | **11.7** | **0** | **0** | **0** | **0** | **0** | **0** | **0** | **0** | **0** | **0** | **0** | **0** | **0** | **0** | **0** | **0** | **0** | **0** | **0** | **0** | **0** | **0** | **0** |
| **Rpo3** | **0** | **0** | **0** | **0** | **0** | **0** | **70.6** | **0** | **0** | **0** | **46.7** | **0** | **33.3** | **0** | **6.7** | **0** | **0** | **0** | **0** | **0** | **0** | **0** | **44.4** | **0** | **85.7** | **0** | **0** | **0** | **0** | **38.5** |
| **Rpo5** | **0** | **0** | **0** | **0** | **0** | **0** | **5.9** | **0** | **0** | **0** | **0** | **0** | **0** | **0** | **0** | **0** | **0** | **0** | **0** | **0** | **0** | **0** | **0** | **0** | **0** | **0** | **0** | **0** | **0** | **0** |
| **Rpo7** | **0** | **0** | **0** | **0** | **0** | **0** | **5.9** | **0** | **0** | **0** | **0** | **60.0** | **0** | **0** | **0** | **0** | **11.1** | **5.3** | **0** | **0** | **0** | **0** | **0** | **0** | **0** | **0** | **0** | **0** | **0** | **7.7** |
| **Rud1** | **0** | **0** | **0** | **0** | **0** | **0** | **0** | **0** | **0** | **0** | **33.3** | **10.0** | **0** | **0** | **26.7** | **0** | **0** | **0** | **0** | **0** | **0** | **0** | **0** | **0** | **0** | **0** | **0** | **0** | **0** | **0** |
| **Rus5** | **0** | **0** | **0** | **0** | **0** | **0** | **0** | **0** | **0** | **11.1** | **0** | **0** | **0** | **0** | **0** | **0** | **0** | **0** | **0** | **0** | **0** | **0** | **0** | **0** | **0** | **0** | **0** | **0** | **0** | **0** |
| **Sar1** | **0** | **0** | **0** | **0** | **0** | **0** | **0** | **0** | **0** | **0** | **0** | **0** | **0** | **0** | **0** | **0** | **22.2** | **0** | **0** | **0** | **0** | **0** | **0** | **0** | **0** | **0** | **0** | **0** | **0** | **0** |
| **Sar3** | **0** | **0** | **0** | **0** | **0** | **0** | **0** | **0** | **0** | **0** | **0** | **0** | **0** | **0** | **0** | **0** | **11.1** | **0** | **0** | **0** | **0** | **0** | **0** | **0** | **0** | **0** | **0** | **0** | **0** | **0** |
| **Stn2** | **0** | **50.0** | **0** | **0** | **0** | **0** | **0** | **0** | **20.0** | **0** | **0** | **10.0** | **0** | **0** | **0** | **0** | **0** | **5.3** | **0** | **81.2** | **0** | **0** | **0** | **0** | **0** | **0** | **0** | **0** | **0** | **0** |
| **Stn4** | **0** | **4.5** | **0** | **11.1** | **0** | **0** | **0** | **0** | **10.0** | **0** | **0** | **10.0** | **0** | **0** | **0** | **0** | **0** | **0** | **0** | **0** | **0** | **0** | **0** | **0** | **0** | **0** | **0** | **0** | **0** | **0** |
| **Stn9** | **0** | **45.5** | **50.0** | **88.9** | **25.0** | **0** | **0** | **0** | **60.0** | **88.9** | **13.3** | **10.0** | **0** | **0** | **0** | **0** | **11.1** | **47.3** | **55.6** | **12.5** | **0** | **0** | **0** | **14.3** | **0** | **100** | **0** | **30.0** | **0** | **53.8** |
| **Szk1** | **0** | **0** | **0** | **0** | **0** | **0** | **0** | **0** | **0** | **0** | **0** | **0** | **0** | **0** | **0** | **0** | **0** | **0** | **0** | **0** | **55.6** | **0** | **0** | **0** | **0** | **0** | **0** | **0** | **0** | **0** |
| **Szk6** | **0** | **0** | **0** | **0** | **0** | **0** | **0** | **0** | **0** | **0** | **0** | **0** | **0** | **0** | **0** | **0** | **0** | **0** | **0** | **0** | **11.1** | **0** | **0** | **0** | **0** | **0** | **0** | **0** | **0** | **0** |
| **Tr10** | **0** | **0** | **8.3** | **0** | **0** | **0** | **0** | **0** | **0** | **0** | **0** | **0** | **0** | **0** | **0** | **0** | **0** | **0** | **0** | **0** | **0** | **0** | **0** | **0** | **0** | **0** | **0** | **0** | **0** | **0** |
