# Supplementary material for: Purifying Selection, Density Blocking and Unnoticed Mitochondrial DNA Diversity in the Red Deer, Cervus elaphus
Source: PLoS One. 2016 Sep 20;11(9):e0163191. doi: 10.1371/journal.pone.0163191 (PMC5029925; doi:10.1371/journal.pone.0163191)
Supplement: S3 Table — Significant values are indicated in bold. (DOCX) [file pone.0163191.s005.docx]

**S3 Table. Genetic differentiation of cr mtDNA between red deer population pairs in Poland, as measured by *Φ*_ST_ (below the diagonal) and *F*_ST_ (above the diagonal).**

| **Population** | **1** | **2** | **3** | **4** | **5** | **6** | **7** | **8** | **9** | **10** | **11** | **12** | **13** | **14** | **15** | **16** | **17** | **18** | **19** | **20** | **21** | **22** | **23** | **24** | **25** | **26** | **27** | **28** | **29** | **30** |
| --- | --- | --- | --- | --- | --- | --- | --- | --- | --- | --- | --- | --- | --- | --- | --- | --- | --- | --- | --- | --- | --- | --- | --- | --- | --- | --- | --- | --- | --- | --- |
| **1** | - | **0.49** | **0.24** | **0.65** | **0.39** | **0.47** | **0.53** | **0.73** | **0.48** | **0.65** | **0.44** | **0.47** | **0.54** | **0.49** | **0.18** | **0.54** | **0.41** | **0.26** | **0.33** | **0.61** | **0.48** | **0.45** | **0.43** | 0.00 | **0.57** | **0.74** | **0.65** | **0.55** | 0.00 | **0.49** |
| **2** | **0.51** | - | **0.23** | **0.28** | **0.22** | **0.39** | **0.46** | **0.65** | 0.03 | **0.29** | **0.33** | **0.32** | **0.46** | **0.41** | **0.29** | **0.46** | **0.30** | **0.15** | **0.17** | **0.15** | **0.41** | **0.39** | **0.36** | **0.41** | **0.53** | **0.38** | **0.56** | **0.39** | **0.56** | **0.23** |
| **3** | 0.06 | **0.30** | - | 0.22 | **0.16** | **0.35** | **0.44** | **0.67** | 0.10 | **0.22** | **0.29** | **0.32** | **0.43** | **0.38** | **0.16** | **0.43** | **0.25** | 0.00 | 0.00 | **0.50** | **0.37** | **0.35** | **0.32** | 0.09 | **0.49** | **0.35** | **0.56** | **0.36** | **0.31** | **0.16** |
| **4** | **0.64** | **0.35** | **0.40** | - | **0.32** | **0.56** | **0.60** | **0.83** | 0.04 | 0.00 | **0.44** | **0.50** | **0.64** | **0.57** | **0.41** | **0.64** | **0.43** | **0.13** | 0.09 | **0.67** | **0.57** | **0.51** | **0.51** | **0.59** | **0.75** | 0.01 | **0.78** | **0.52** | **0.78** | 0.18 |
| **5** | **0.44** | **0.30** | **0.26** | **0.55** | - | **0.23** | **0.35** | **0.57** | 0.12 | **0.32** | **0.21** | **0.22** | **0.26** | **0.25** | **0.09** | **0.33** | **0.15** | **0.11** | 0.10 | **0.43** | **0.25** | **0.25** | **0.20** | **0.26** | **0.42** | **0.47** | **0.47** | **0.22** | **0.47** | **0.17** |
| **6** | **0.75** | **0.78** | **0.71** | **0.83** | **0.70** | - | **0.42** | **0.69** | **0.34** | **0.56** | **0.31** | **0.33** | **0.41** | **0.35** | **0.22** | **0.22** | **0.26** | **0.29** | **0.31** | **0.53** | **0.34** | 0.11 | **0.18** | **0.40** | **0.51** | **0.69** | **0.33** | **0.43** | **0.56** | **0.36** |
| **7** | **0.63** | **0.60** | **0.54** | **0.89** | **0.48** | **0.84** | - | **0.69** | **0.43** | 0.60 | 0.10 | **0.40** | **0.34** | **0.44** | **0.28** | **0.50** | **0.36** | **0.38** | **0.41** | **0.57** | **0.44** | **0.41** | 0.10 | **0.49** | 0.00 | **0.69** | **0.60** | **0.51** | **0.60** | **0.24** |
| **8** | **0.78** | **0.77** | **0.74** | **0.98** | **0.58** | **0.90** | **0.90** | - | **0.68** | **0.83** | **0.62** | **0.67** | **0.23** | **0.61** | **0.55** | **0.74** | **0.63** | **0.58** | **0.67** | **0.77** | **0.69** | **0.63** | **0.65** | **0.75** | **0.81** | **0.89** | **0.83** | **0.19** | **0.83** | **0.67** |
| **9** | **0.50** | 0.01 | **0.25** | 0.17 | **0.28** | **0.76** | **0.68** | **0.85** | - | 0.06 | **0.27** | **0.28** | **0.42** | **0.37** | **0.24** | **0.42** | **0.22** | 0.02 | 0.00 | **0.38** | **0.36** | **0.34** | **0.30** | **0.35** | **0.51** | 0.19 | **0.56** | **0.32** | **0.56** | 0.08 |
| **10** | **0.64** | **0.36** | **0.40** | 0.00 | **0.55** | **0.83** | **0.89** | **0.98** | 0.19 | - | **0.44** | **0.50** | **0.64** | **0.57** | **0.41** | **0.64** | **0.43** | 0.13 | 0.09 | **0.67** | **0.57** | **0.51** | **0.51** | **0.59** | **0.75** | 0.01 | **0.78** | **0.52** | **0.78** | 0.18 |
| **11** | **0.46** | **0.29** | **0.28** | **0.53** | **0.20** | **0.76** | **0.29** | **0.75** | **0.29** | **0.53** | - | **0.28** | **0.28** | **0.34** | **0.11** | **0.39** | **0.24** | **0.24** | **0.25** | **0.48** | **0.33** | **0.32** | 0.09 | **0.36** | 0.14 | **0.54** | **0.50** | **0.38** | **0.50** | 0.13 |
| **12** | **0.45** | **0.30** | **0.28** | **0.55** | 0.17 | **0.73** | **0.24** | **0.70** | **0.30** | **0.55** | 0.06 | - | **0.41** | **0.36** | **0.20** | **0.41** | **0.20** | **0.23** | **0.28** | **0.47** | **0.35** | **0.33** | **0.29** | **0.39** | **0.50** | **0.63** | **0.55** | **0.42** | **0.55** | **0.30** |
| **13** | **0.56** | **0.56** | **0.46** | **0.83** | 0.13 | **0.76** | **0.54** | **0.40** | **0.60** | **0.83** | **0.40** | **0.26** | - | **0.35** | **0.28** | **0.50** | **0.35** | **0.36** | **0.40** | **0.59** | **0.43** | **0.40** | **0.27** | **0.49** | **0.44** | **0.76** | **0.64** | 0.09 | **0.64** | **0.36** |
| **14** | **0.66** | **0.70** | **0.61** | **0.73** | **0.58** | **0.23** | **0.73** | **0.82** | **0.66** | **0.73** | **0.66** | **0.61** | **0.64** | - | **0.25** | **0.30** | **0.29** | **0.32** | **0.34** | **0.54** | **0.21** | **0.33** | **0.32** | **0.42** | **0.52** | **0.69** | **0.57** | **0.37** | **0.57** | **0.39** |
| **15** | **0.19** | **0.28** | 0.08 | **0.45** | 0.08 | **0.69** | **0.32** | **0.57** | **0.28** | **0.45** | 0.09 | **0.13** | **0.24** | **0.59** | - | **0.30** | **0.14** | **0.13** | **0.16** | **0.40** | **0.24** | **0.23** | **0.16** | 0.12 | **0.31** | **0.51** | **0.41** | **0.31** | **0.24** | **0.24** |
| **16** | **0.80** | **0.83** | **0.77** | **0.88** | **0.79** | 0.08 | **0.89** | **0.93** | **0.82** | **0.88** | **0.82** | **0.80** | **0.83** | **0.25** | **0.76** | - | **0.35** | **0.36** | **0.40** | **0.59** | **0.36** | 0.03 | 0.20 | **0.49** | **0.60** | **0.76** | 0.11 | **0.52** | **0.64** | **0.44** |
| **17** | **0.49** | **0.39** | **0.32** | **0.56** | **0.24** | **0.73** | **0.34** | **0.74** | **0.36** | **0.56** | **0.19** | 0.00 | **0.32** | **0.61** | **0.21** | **0.80** | - | **0.19** | **0.16** | **0.46** | **0.28** | **0.27** | **0.22** | **0.31** | **0.43** | **0.56** | **0.49** | **0.35** | **0.46** | **0.25** |
| **18** | **0.24** | **0.15** | 0.00 | **0.18** | **0.16** | **0.71** | **0.43** | **0.65** | 0.07 | **0.18** | **0.17** | **0.17** | **0.39** | **0.62** | **0.09** | **0.77** | **0.22** | - | 0.00 | **0.39** | **0.31** | **0.30** | **0.26** | 0.14 | **0.41** | **0.23** | **0.47** | **0.28** | **0.32** | **0.10** |
| **19** | **0.29** | **0.17** | 0.02 | 0.15 | **0.17** | **0.70** | **0.53** | **0.76** | 0.05 | 0.15 | **0.18** | **0.17** | **0.44** | **0.60** | 0.11 | **0.78** | 0.18 | 0.00 | - | **0.48** | **0.33** | **0.32** | **0.28** | 0.19 | **0.48** | **0.24** | **0.54** | **0.31** | **0.42** | 0.08 |
| **20** | **0.61** | **0.22** | **0.51** | **0.81** | **0.54** | **0.82** | **0.78** | **0.90** | **0.46** | **0.81** | **0.52** | **0.52** | **0.73** | **0.73** | **0.41** | **0.87** | **0.62** | **0.41** | **0.51** | - | **0.54** | **0.50** | **0.49** | **0.59** | **0.68** | **0.77** | **0.70** | **0.59** | **0.70** | **0.50** |
| **21** | **0.85** | **0.87** | **0.83** | **0.97** | **0.87** | **0.62** | **0.95** | **0.98** | **0.89** | **0.97** | **0.88** | **0.87** | **0.92** | 0.14 | **0.80** | **0.60** | **0.87** | **0.81** | **0.85** | **0.93** | - | **0.33** | **0.31** | **0.41** | **0.52** | **0.69** | **0.57** | **0.45** | **0.57** | **0.38** |
| **22** | **0.72** | **0.75** | **0.69** | **0.76** | **0.68** | 0.02 | **0.77** | **0.84** | **0.72** | **0.76** | **0.72** | **0.69** | **0.72** | **0.27** | **0.68** | 0.02 | **0.70** | **0.70** | **0.68** | **0.77** | **0.52** | - | **0.13** | **0.39** | **0.48** | **0.61** | 0.04 | **0.42** | **0.51** | **0.36** |
| **23** | **0.52** | **0.56** | **0.45** | **0.58** | **0.39** | 0.08 | **0.54** | **0.71** | **0.49** | **0.58** | **0.47** | **0.41** | **0.45** | 0.14 | **0.41** | 0.23 | **0.42** | **0.47** | **0.42** | **0.59** | **0.49** | **0.15** | - | **0.35** | 0.14 | **0.64** | **0.31** | **0.40** | **0.51** | **0.19** |
| **24** | 0.00 | **0.51** | 0.04 | **0.75** | **0.43** | **0.72** | **0.71** | **0.87** | **0.53** | **0.75** | **0.46** | **0.45** | **0.60** | **0.61** | 0.13 | **0.79** | **0.50** | **0.23** | 0.29 | **0.64** | **0.88** | **0.69** | **0.44** | - | **0.55** | **0.74** | **0.64** | **0.49** | 0.00 | **0.38** |
| **25** | **0.43** | **0.50** | **0.32** | **0.83** | **0.31** | **0.74** | 0.04 | **0.88** | **0.54** | **0.83** | 0.16 | **0.14** | **0.42** | **0.61** | 0.13 | **0.82** | **0.23** | **0.27** | **0.33** | **0.69** | **0.92** | **0.69** | **0.38** | **0.45** | - | **0.88** | **0.75** | **0.61** | **0.71** | **0.30** |
| **26** | **0.65** | **0.37** | **0.42** | 0.01 | **0.59** | **0.84** | **0.91** | **0.98** | 0.21 | 0.01 | **0.56** | **0.58** | **0.85** | **0.75** | **0.47** | **0.89** | **0.59** | **0.19** | **0.17** | **0.83** | **0.98** | **0.77** | **0.60** | **0.77** | **0.86** | - | **0.89** | **0.67** | **0.89** | **0.31** |
| **27** | **0.81** | **0.84** | **0.79** | **0.89** | **0.80** | 0.11 | **0.90** | **0.94** | **0.83** | **0.89** | **0.83** | **0.81** | **0.85** | **0.43** | **0.77** | 0.06 | **0.81** | **0.78** | **0.79** | **0.88** | **0.75** | 0.00 | **0.25** | **0.81** | **0.83** | **0.90** | - | **0.65** | **0.78** | **0.56** |
| **28** | **0.53** | **0.44** | **0.37** | **0.64** | 0.01 | **0.74** | **0.58** | **0.36** | **0.43** | **0.64** | **0.36** | **0.23** | 0.07 | **0.63** | **0.22** | **0.82** | **0.27** | **0.27** | **0.29** | **0.66** | **0.89** | **0.71** | **0.46** | **0.54** | **0.46** | **0.67** | **0.83** | - | **0.65** | **0.36** |
| **29** | 0.04 | **0.68** | **0.27** | **0.89** | **0.63** | **0.79** | **0.82** | **0.92** | **0.72** | **0.89** | **0.64** | **0.62** | **0.74** | **0.69** | **0.32** | **0.84** | **0.65** | **0.43** | **0.52** | **0.79** | **0.93** | **0.74** | **0.54** | 0.00 | **0.65** | **0.90** | **0.86** | **0.70** | - | **0.56** |
| **30** | **0.49** | **0.24** | **0.25** | 0.34 | **0.20** | **0.77** | **0.44** | **0.80** | **0.16** | 0.34 | **0.11** | 0.10 | **0.46** | **0.66** | **0.20** | **0.83** | 0.14 | 0.08 | 0.04 | **0.59** | **0.89** | **0.73** | **0.48** | **0.53** | **0.31** | **0.36** | **0.84** | **0.34** | **0.70** | - |

Significant values are indicated in bold.
