# Supplementary material for: Purifying Selection, Density Blocking and Unnoticed Mitochondrial DNA Diversity in the Red Deer, Cervus elaphus
Source: PLoS One. 2016 Sep 20;11(9):e0163191. doi: 10.1371/journal.pone.0163191 (PMC5029925; doi:10.1371/journal.pone.0163191)
Supplement: S4 Table — (DOCX) [file pone.0163191.s006.docx]

**S4 Table. Effects of abiotic and climatic factors on genetic differentiation (as measured by *F*_ST_ values) among 30 red deer populations in Poland based on cr mtDNA.**

| Factor | Marginal tests | | Conditional tests | | Sequential tests | |
| --- | --- | --- | --- | --- | --- | --- |
|  | % var | *P* | % var | *P* | % var | *P* |
| Latitude | 12.1 | <0.01 | - | - | 2.5 | ns |
| Longitude | 9.9 | <0.05 | - | - | 8.6 | <0.05 |
| Snow cover depth | 13.3 | <0.01 | 5.5 | ns | 0.5 | ns |
| Days with snow cover | 14.5 | <0.01 | 6.3 | ns | 0.1 | ns |
| Frost days | 12.4 | <0.01 | 8.9 | <0.05 | 5.5 | ns |
| Temperature in January | 10.3 | <0.05 | 3.9 | ns | 3.4 | ns |
| Annual rainfall | 14.1 | <0.01 | 6.9 | ns | 1.4 | ns |
| Summer season | 13.6 | <0.01 | 7.3 | <0.05 | 1.0 | ns |
| The Elsterian | 17.0 | <0.001 | 8.0 | <0.05 | 17.0 | 0.001 |

% var – percentage of genetic variation explained by the particular variable; P – probability values; ns – non significant; the Elsterian means the southernmost ice sheet limit during the maximum extension in the Pleistocene.
